# Complex Magnetic Order in Topochemically Reduced Rh(I)/Rh(III) LaM_0.5_Rh_0.5_O_2.25_ (M = Co, Ni) Phases

**DOI:** 10.1021/acs.inorgchem.2c02747

**Published:** 2022-09-21

**Authors:** Zheying Xu, Pardeep K. Thakur, Tien-Lin Lee, Anna Regoutz, Emmanuelle Suard, Inés Puente-Orench, Michael A. Hayward

**Affiliations:** † Department of Chemistry, University of Oxford, Inorganic Chemistry Laboratory, South Parks Road, Oxford OX1 3QR, U.K.; ‡ Department of Chemistry, University College London, 20 Gordon Street, London WC1H 0AJ, U.K.; § Diamond Light Source Ltd., Diamond House, Harwell Science and Innovation Campus, Didcot OX11 0DE, U.K.; ∥ Institut Laue-Langevin - 71 avenue des Martyrs, 38000 Grenoble, France

## Abstract

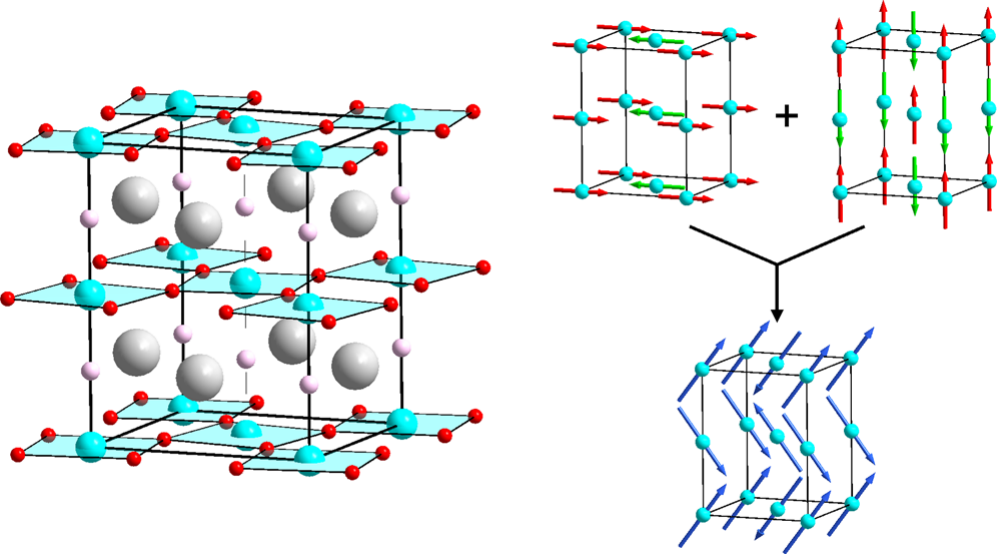

Topochemical reduction of the cation-disordered perovskite oxides LaCo_0.5_Rh_0.5_O_3_ and LaNi_0.5_Rh_0.5_O_3_ with Zr yields the partially anion-vacancy ordered phases LaCo_0.5_Rh_0.5_O_2.25_ and LaNi_0.5_Rh_0.5_O_2.25_, respectively. Neutron diffraction and Hard X-ray photoelectron spectroscopy (HAXPES) measurements reveal that the anion-deficient phases contain Co^1+^/Ni^1+^ and a 1:1 mixture of Rh^1+^ and Rh^3+^ cations within a disordered array of apex-linked MO_4_ square-planar and MO_5_ square-based pyramidal coordination sites. Neutron diffraction data indicate that LaCo_0.5_Rh_0.5_O_2.25_ adopts a complex antiferromagnetic ground state, which is the sum of a C-type ordering (mM_5_^+^) of the *xy*-components of the Co spins and a G-type ordering (mΓ_1_^+^) of the *z*-components of the Co spins. On warming above 75 K, the magnitude of the mΓ_1_^+^ component declines, attaining a zero value by 125 K, with the magnitude of the mM_5_^+^ component remaining unchanged up to 175 K. This magnetic behavior is rationalized on the basis of the differing d-orbital fillings of the Co^1+^ cations in MO_4_ square-planar and MO_5_ square-based pyramidal coordination sites. LaNi_0.5_Rh_0.5_O_2.25_ shows no sign of long-range magnetic order at 2 K – behavior that can also be explained on the basis of the d-orbital occupation of the Ni^1+^ centers.

## Abstract 2

Topochemical reduction of LaM0.5Rh0.5O3 (M = Co, Ni) yields LaM0.5Rh0.5O2.25, containing Co^1+^/Ni^1+^ and a 1:1 disordered array of Rh^1+^ and Rh^3+^. LaCo_0.5_Rh_0.5_O_2.25_ adopts an antiferromagnetic ground state, which is the sum of a C-type ordering of the *xy*-components of the Co^1+^ spins and a G-type ordering of the *z*-components of the Co^1+^ spins.

## Introduction

The wide range of both chemical and physical behaviors exhibited by transition metal oxides has led to extensive and enduring interest in the chemistry and physics of these compounds. A particular attraction of many transition-metal oxide systems is that their physical and chemical behavior can be rationally tuned by modifying the configuration of the metal d-states (electron count, orbital occupation) via chemical substitution or structural modification. However, most transition metals only exhibit a limited range of thermodynamically stable oxidation-state/coordination-geometry combinations in oxide environments, limiting this approach. For example, late 4d and 5d transition metals (Ru, Rh, Re, Os, Ir) strongly disfavor low oxidation states (e.g., M^1+^ or M^2+^) when located in extended oxide frameworks.^1^ The thermodynamic instability of these low oxidation states can be attributed to the high atomization energies of the elements and relatively low cumulative ionization energies of higher-oxidation states (M^n+^*n* ≥ 3), which combine to make the M^2+^ oxidation state unstable with respect to disproportionation when bonded to good ligands such as oxide ions. This effect can be illustrated by comparing the stable binary oxides of the late 4d/5d transition metals, which have the lowest oxygen content, with their 3d transition metal analogues: RuO_2_ and OsO_2_ compared to FeO; Rh_2_O_3_ and IrO_2_ compared to CoO.^1^

Topochemical reduction by anion deintercalation allows the preparation of metastable phases containing late 4d/5d transition metal cations with divalent charges, such as Ru^2+^ and Ir^2+^,^2−6^ enabling their electronic and magnetic behaviors to be studied. Recently, when trying to extend this chemistry to rhodium via the preparation of the Rh^2+^-containing extended oxides LaSrCo_0.5_Rh_0.5_O_3.25_ and LaSrNi_0.5_Rh_0.5_O_3.25_, we observed a further valence instability in which d^7^ Rh^2+^ disproportionates into d^8^ Rh^1+^ and d^6^ Rh^3+^, driven by the presence of square planar and square-based pyramidal coordination sites.^7^ This behavior is analogous to the disproportionation of d^7^ Pd^3+^ in KPd_2_O_3_ (better thought of as K_2_Pd_3_^II^Pd^IV^O_6_)^8^ or d^7^ Pt^3+^ in CdPt_3_O_6_ (better thought of as CdPt^II^Pt_2_^IV^O_6_),^9^ and in the case of the LaSrM_0.5_Rh_0.5_O_4–*x*_ reduced phases, it appears to preferentially “select” the composition of the anion-deficient phases. To further explore this behavior, we have investigated the topochemical reduction of the analogous perovskite oxides, LaCo_0.5_Rh_0.5_O_3_ and LaNi_0.5_Rh_0.5_O_3_, which we report here.

## Experimental Section

### Preparation of LaM_0.5_Rh_0.5_O_3_ (M = Co, Ni)

Samples of LaM_0.5_Rh_0.5_O_3_ (M = Co, Ni) were prepared by a ceramic method. Suitable quantities of La_2_O_3_ (99.999%, dried at 900 °C), Rh_2_O_3_ (99.998%, dried at 850 °C), Co_3_O_4_ (99.99%), or elemental Ni (99.996%) were ground together using an agate pestle and mortar. The mixed powders were heated in air at a rate of 5 °C min^–1^ to 1000 °C in alumina crucibles to oxidize the metals. After heating, the powders were pressed into pellets and then heated in air for 48 h periods at 1000, 1200, and then 1250 °C. X-ray powder diffraction data collected from the rhodium-containing perovskite phases yielded lattice parameters in agreement with previously reported literature values, as detailed in the Supporting Information.^10,11^

### Reduction of LaM_0.5_Rh_0.5_O_3_ (M = Co, Ni)

Samples of LaM_0.5_Rh_0.5_O_3_ (M = Co, Ni) were reduced using a zirconium getter.^12^ Samples to be reduced were sealed in evacuated silica ampoules along with a glass “thimble” containing 2 mole equivalents of powdered zirconium, such that the two powders shared an atmosphere but were not in physical contact. Small-scale test reactions were performed in which ∼200 mg of the rhodium perovskite samples were heated at a rate of 1 °C min^–1^ to temperatures in the range 350–500 °C and held there for 3 periods of 5 days to assess reactivity. This revealed that the optimum temperatures for reduction are LaCo_0.5_Rh_0.5_O_3_: 400 °C and LaNi_0.5_Rh_0.5_O_3_: 420 °C. Samples of LaCo_0.5_Rh_0.5_O_3*–x*_ and LaNi_0.5_Rh_0.5_O_3*–x*_ studied by neutron diffraction were prepared by heating ∼2g of the corresponding oxide with 2 mole equivalents of Zr at the optimum reduction temperature for 3 periods of 5 days, with samples being reground and the Zr replaced between heating periods.

### Characterization

Reaction progress and initial structural characterization were performed using laboratory X-ray powder diffraction (PXRD) data collected using a PANalytical X′pert diffractometer incorporating an X′celerator position-sensitive detector (monochromatic Cu Kα1 radiation). High-resolution synchrotron X-ray powder diffraction (SXRD) data were collected using the I11 instrument at the Diamond Light Source Ltd. Diffraction patterns were collected using Si-calibrated X-rays with an approximate wavelength of 0.825 Å from samples, sealed in 0.3 mm diameter borosilicate glass capillaries. Neutron powder diffraction (NPD) data were collected at room temperature using the D2B diffractometer (λ = 1.594 Å), and data at low temperature were collected using the D1B diffractometer (λ = 2.52 Å) at the ILL neutron source, from samples contained within vanadium cans sealed under an inert atmosphere. Rietveld refinement of powder diffraction data was performed using TOPAS Academic (V6).^13^

Thermogravimetric analysis (TGA) measurements were performed by heating powder samples at a rate of 5 °C min^–1^ under flowing 10%H_2_/90%N_2_, using a Mettler-Toledo MX1 thermogravimetric microbalance, and then cooling to 25 °C. DC magnetization data were collected using a Quantum Design MPMS SQUID magnetometer from samples contained in gelatin capsules.

Hard X-ray photoelectron spectroscopy (HAXPES) measurements were conducted at beamline I09 of the Diamond Light Source, UK.^14^ A photon energy of 5.9 keV was selected using a double-crystal Si (111) monochromator in combination with a Si (004) channel-cut crystal post-monochromator. The end station of the beamline is equipped with a Scienta Omicron EW4000 hemispherical analyzer with a ±28° acceptance angle. All spectra were collected in grazing incidence and near-normal emission, and the sample was mounted on conducting carbon tape.

## Results and Discussion

### Chemical and Structural Characterization of LaM_0.5_Rh_0.5_O_3*–x*_ (M = Co, Ni)

Heating LaM_0.5_Rh_0.5_O_3*–x*_ reduced samples under a 10%H_2_:90%N_2_ atmosphere led to decomposition to the corresponding mixtures of La_2_O_3_, Rh, and Co or Ni. TGA data collected during this process indicated mass losses consistent with compositions of LaCo_0.5_Rh_0.5_O_2.25(1)_ and LaNi_0.5_Rh_0.5_O_2.25(1)_, as described in detail in the Supporting Information.

**Figure 1:**
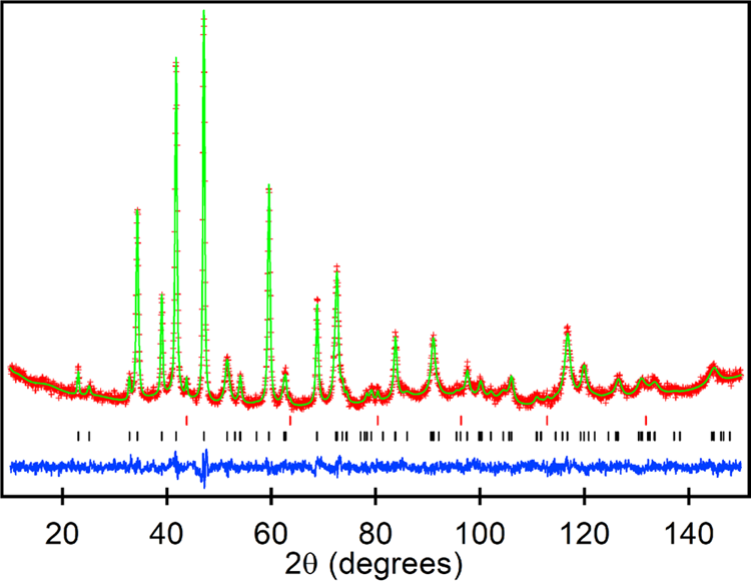
Observed, calculated, and difference plots from the structural refinement of LaCo_0.5_Rh_0.5_O_2.25_ against NPD data collected at room temperature using instrument D2B. Black and red tick marks indicate peak positions for the majority phase and contributions from the vanadium sample holder, respectively.

NPD data collected from LaCo_0.5_Rh_0.5_O_2.25_ at room temperature could be indexed using a body-centered unit cell (*a* = 5.6492(2) Å, *c* = 7.3374(7) Å) with reflection conditions consistent with the *I*4/*mcm* (#140) space group. A structural model was constructed based on a Co/Rh, B-site disordered perovskite phase with an *a*^0^*a*^0^*c*^–^ tilting distortion, analogous to the reported structure of SrTi_0.5_Zr_0.5_O_3_.^15^ This model was refined against the NPD data. During the refinement, the occupancies of the oxide ion sites were allowed to vary, and it was observed that the 8*h* site remained fully occupied within error, but the occupancy of the 4*a* site declined to 0.25(1) consistent with the composition of the phase determined from TGA data. All other atomic positional and displacement parameters were allowed to vary, yielding a model that gave a good fit to the NPD data as shown in Figure 1 and detailed in Table S1, with selected bond lengths and angles in Table S3 in the Supporting Information.

**Figure 2:**
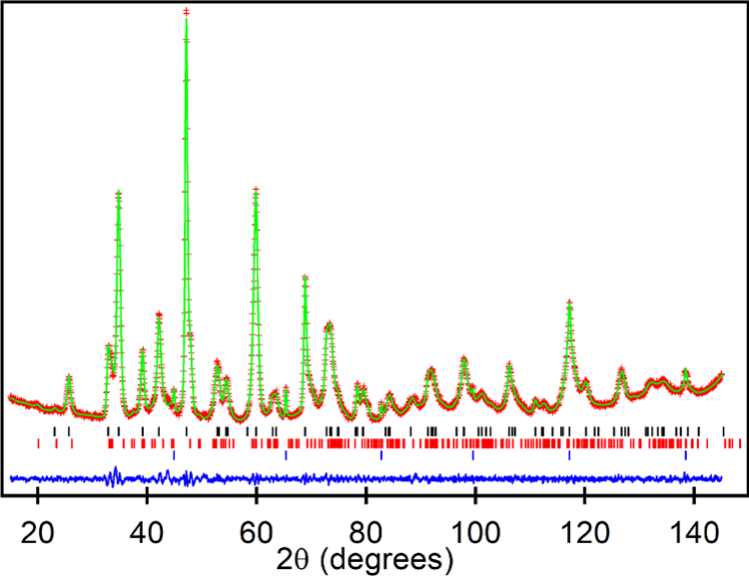
Observed, calculated, and difference plots from the structural refinement of LaNi_0.5_Rh_0.5_O_2.25_ against NPD data collected at room temperature using instrument D2B. Black and red and blue tick marks indicate peak positions for the majority phase and a LaNi_0.5_Rh_0.5_O_3_ secondary phase and contributions from the vanadium sample holder, respectively.

NPD data collected from LaNi_0.5_Rh_0.5_O_2.25_ at room temperature could also be indexed using a body-centered tetragonal unit cell (*a* = 5.6407(1) Å, *c* = 7.1794(7) Å) with reflection conditions consistent with the *I*4/*mcm* (#140) space group. A structural model analogous to that used for LaCo_0.5_Rh_0.5_O_2.25_ was refined against these data. Refinement of the anion site occupancies revealed that the 8*h* sites remained fully occupied, while the 4*a* sites yielded an occupancy of 0.24(2), consistent with the composition determined by TGA. Close inspection of the NPD data collected from LaNi_0.5_Rh_0.5_O_2.25_ revealed additional diffraction peaks not indexed by the body-centered tetragonal cell, but which could be modeled using a second perovskite phase corresponding to 9.2 wt % unreduced LaNi_0.5_Rh_0.5_O_3_. This two-phase model gave a good fit to the NPD data as shown in Figure 2 and detailed in Table S2, with selected bond lengths and angles in Table S3 in the Supporting Information.

**Figure 3:**
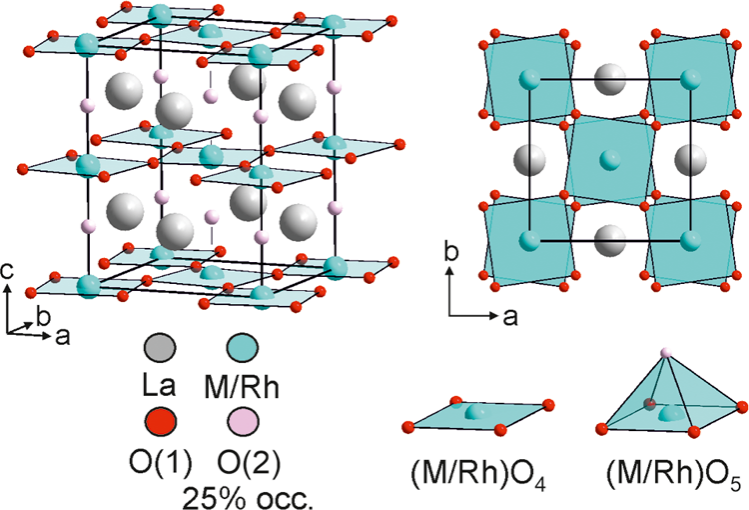
Structure of LaM_0.5_Rh_0.5_O_2.25_ (M = Co, Ni) and the local (M/Rh)O_*x*_ coordination polyhedra.

The anion-deficient perovskite structures adopted by LaCo_0.5_Rh_0.5_O_2.25_ and LaNi_0.5_Co_0.5_O_2.25_ are shown in Figure 3. Topochemical reduction with Zr has removed 75% of the O(2) apical oxide ions from the parent LaM_0.5_Rh_0.5_O_3_ phases to yield materials in which the apex-linked (M/Rh)O_6_ units have been converted into a 1:1 disordered array of (M/Rh)O_4_ square planes and (M/Rh)O_5_ square-based pyramids, as shown in Figure 3. Thus, it can be seen that the topochemical reduction of LaM_0.5_Rh_0.5_O_3_ (M = Co, Ni) phases to LaM_0.5_Rh_0.5_O_2.25_ phases is structurally analogous to the reduction of LaSrM_0.5_Rh_0.5_O_4_*n* = 1 Ruddlesden–Popper phases to LaSrM_0.5_Rh_0.5_O_3.25_ phases, as these latter reduced phases also contain disordered arrays of (M/Rh)O_4_ square planes and (M/Rh)O_5_ square-based pyramids.^7^ Indeed, comparison of the bond lengths of the LaM_0.5_Rh_0.5_O_2.25_ phases (Table S3) with those of the LaSrM_0.5_Rh_0.5_O_3.25_ materials reveals that the transition-metal coordination environments in corresponding phases are remarkably similar (<(Rh/Co)-O_eq_> = 2.065 Å, <(Rh/Co)-O_ax_> = 1.802 Å; <(Rh/Ni)-O_eq_> = 2.059 Å, <(Rh/Ni)-O_ax_> = 1.790 Å),^7^ suggesting that the corresponding reduced phases have the same combination of average transition-metal oxidations states: Co^1+^/Ni^1+^ (seen previously in a number of topochemically reduced phases)^3,4,6,16−18^ combined with “Rh^2+^.”

**Figure 4:**
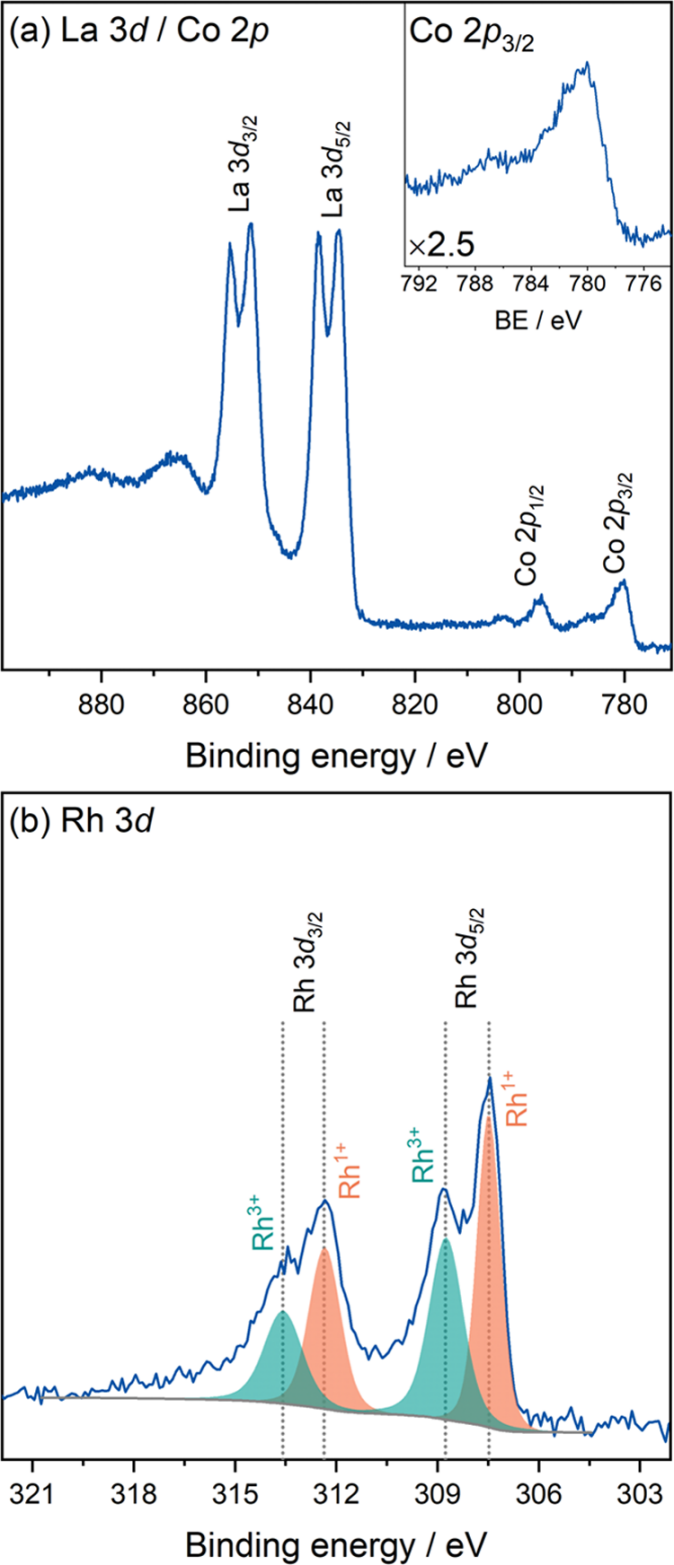
HAXPES core-level spectra of LaCo_0.5_Rh_0.5_O_2.25_, including (a) La 3d/Co 2p and (b) Rh 3d. The inset in panel (a) shows a magnified (×2.5) view of the Co 2p_3/2_ line.

Given that the nominal Rh^2+^ centers in LaSrCo_0.5_Rh_0.5_O_3.25_ and LaSrNi_0.5_Rh_0.5_O_3.25_ are observed to disproportionate into Rh^1+^ and Rh^3+^, driven by the presence of square-planar and square-pyramidal coordination sites, it appeared likely that the nominal Rh^2+^ centers in the LaM_0.5_Rh_0.5_O_2.25_ phases would also undergo a similar disproportionation. To investigate this possibility, HAXPES was used to explore the chemical state of the LaCo_0.5_Rh_0.5_O_2.25_ sample. The survey spectrum (Figure S5 in the Supporting Information) showed all expected core-level features as well as a small contribution from adventitious carbon. The main La 3d and Co 2p core-level spectra (Figure 4a) are commensurate with the oxide environments present in LaCo_0.5_Rh_0.5_O_2.25_. The Rh 3d core level (Figure 4b) confirms the disproportionation of Rh^2+^ centers, showing a clear split into a lower binding energy (BE) Rh^1+^ (at 307.5 and 312.4 eV) and a higher BE Rh^3+^ component (at 308.8 and 313.6 eV) for both the Rh 3d_5/2_ and 3d_3/2_ components. The main advantage of using HAXPES instead of conventional soft X-ray photoelectron spectroscopy (SXPS) is the increase in probing depth and therefore the bulk sensitivity of the measurements, excluding that this is purely a surface effect.^19^ The O 1s spectrum (Figure S5 in the Supporting Information) emphasizes the probing depth with only a low contribution from surface hydroxide species, in particular, for a powder mixed oxide sample. Although the peak fit of the Rh 3d core level is complicated due to the complex background displayed, fits of the Rh^1+^ and Rh^3+^ components result in line shapes matching previous observations for related oxides, including a slight increase in peak width for the 3d_3/2_ component from Coster–Kronig broadening.^7,20,21^ Voigt functions were used for all fitted peaks with the Gaussian and Lorentzian contributions allowed to vary between 20 and 30% as the tails are not well defined due to the complex background. The full width at half-maximum (FWHM) for the Rh^1+^ component in the Rh 3d spectrum is 0.8 eV for 3d_5/2_ and 1.1 eV for 3d_3/2_, with an area ratio of 3.0:2.1. Based on the areas resulting from the peak fit, an approximate 1:1 ratio of the Rh^1+^ and Rh^3+^ states is observed. The presence of Rh^+1^ states is further corroborated by the valence spectrum (Figure S5 in the Supporting Information), which shows a clear contribution from these states just below the Fermi level (E_F_).

### Magnetic Characterization of LaM_0.5_Rh_0.5_O_3*–x*_ (M = Co, Ni)

Magnetization data collected from LaCo_0.5_Rh_0.5_O_2.25_ and LaNi_0.5_Rh_0.5_O_2.25_ indicate that the samples prepared of these materials contain small amounts of ferromagnetic Co and Ni, respectively. Thus, magnetization data were collected using a “ferromagnetic subtraction” technique, described in detail in the Supporting Information, which utilizes the observation that the magnetization of Co and Ni saturate in applied fields greater than 2T.

**Figure 5:**
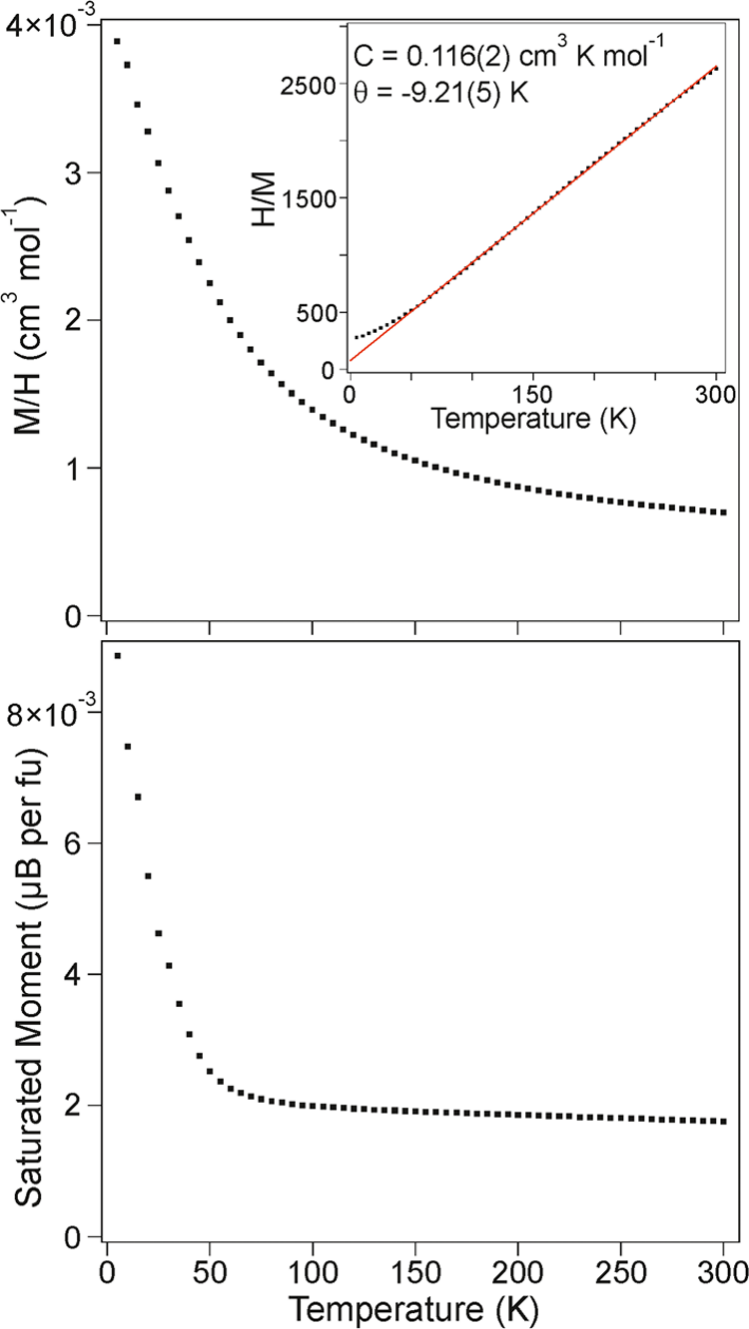
Paramagnetic susceptibility (top) and saturated ferromagnetic moment (bottom) of LaNi_0.5_Rh_0.5_O_2.25_ plotted as a function of temperature. The inset shows fit to the Curie–Weiss law in the range 70 < *T*/K < 300.

The paramagnetic susceptibility of LaNi_0.5_Rh_0.5_O_2.25_ (Figure 5) can be fit by the Curie–Weiss law (χ = C/(*T*–θ)) in the temperature range 70 < *T*/K < 300 to yield values of *C* = 0.116(2) cm^3^ K mol^–1^ and θ = −9.21 K. The observed value of the Curie constant is broadly consistent with the value expected for a combination of *S* = 1/2 Ni^1+^ and *S* = 0, Rh^1+^/*S* = 0, Rh^3+^ (*C*_expected_ = 0.1875 cm^3^ K mol^–1^). Below 70 K, the paramagnetic susceptibility deviates from the Curie–Weiss law and there is a sharp increase in the saturated ferromagnetic moment of the sample, suggesting the onset of magnetic order. However, neutron powder diffraction data collected from LaNi_0.5_Rh_0.5_O_2.25_ at 2 K show no evidence of long-range magnetic order, as shown in Figure S7 in the Supporting Information.

**Figure 6:**
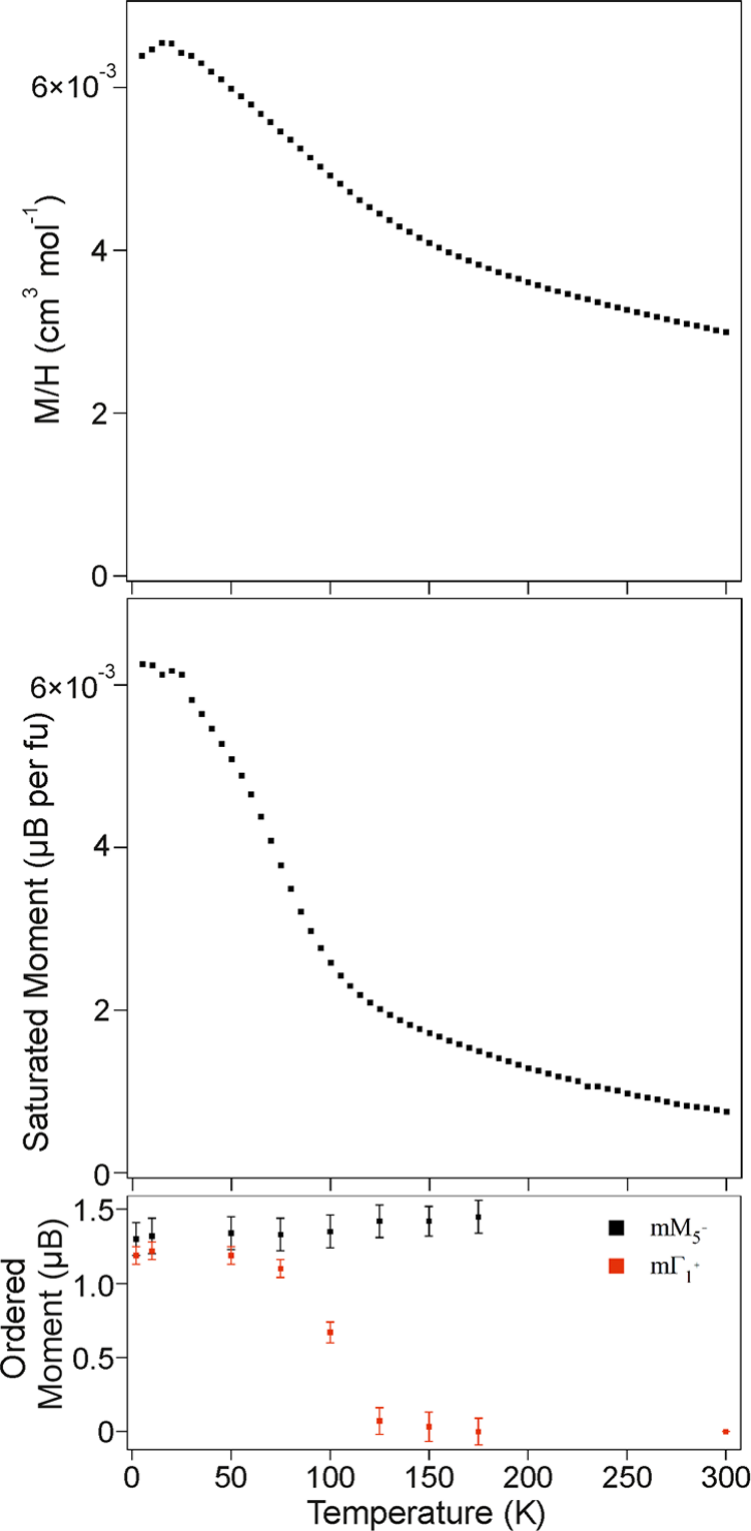
Paramagnetic susceptibility (top) and saturated ferromagnetic moment (middle) of LaCo_0.5_Rh_0.5_O_2.25_ plotted as a function of temperature. Ordered magnetic moment (bottom) extracted from fits to NPD data.

The magnetic behavior of LaCo_0.5_Rh_0.5_O_2.25_ is more complex. The paramagnetic susceptibility of LaCo_0.5_Rh_0.5_O_2.25_ (Figure 6) follows the mathematical form of the Curie–Weiss law (χ = *C*/(*T–*θ)) in the range 210 < *T*/K < 300, as shown in Figure S8 in the Supporting information. However, the parameters extracted by fitting the data (*C* = 1.77 cm^3^ K mol^–1^, θ = −292 K) are much larger than would be expected from a combination of *S* = 1 Co^1+^ centers and *S* = 0, Rh^1+^/*S* = 0, Rh^3+^ centers (*C*_expected_ = 0.5 cm^3^ K mol^–1^), indicating that a “simple paramagnetic” description is not valid for LaCo_0.5_Rh_0.5_O_2.25_ in this temperature range. On cooling, the magnetic susceptibility of LaCo_0.5_Rh_0.5_O_2.25_ exhibits a weak maximum at *T* ∼ 15 K, and the saturated ferromagnetic moment of the sample shows a sharp increase below 125 K, suggesting a transition to a magnetically ordered state. Magnetization-field isotherms collected from LaCo_0.5_Rh_0.5_O_2.25_ at 5 K after cooling from 300 K in an applied field of 50,000 Oe (Figure S9) show weak hysteresis and are displaced from the origin, indicating a glassy component to the magnetic state below 30 K.

**Figure 7:**
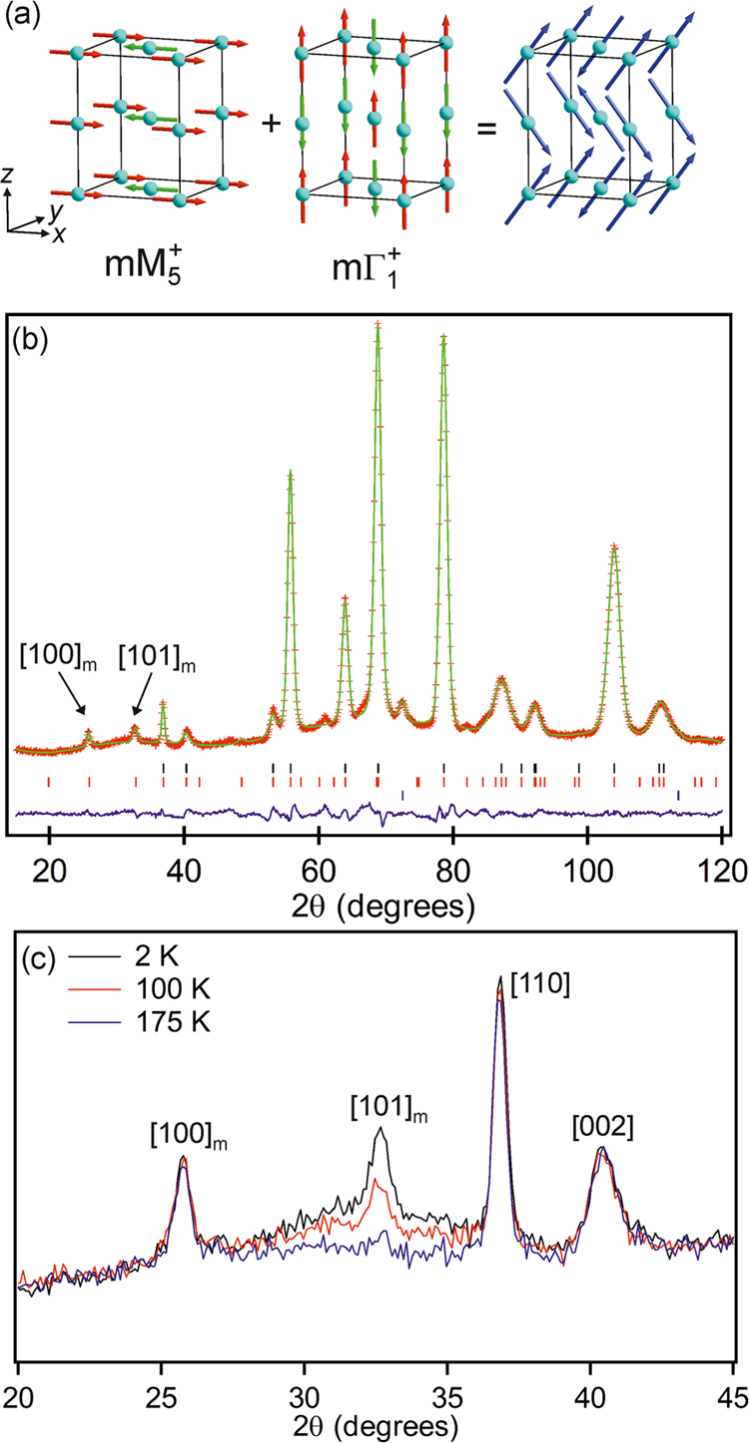
(a) mM_5_^+^ and mΓ_1_^+^ symmetry magnetic orderings on LaCo_0.5_Rh_0.5_O_2.25_. (b) Observed, calculated, and difference plots from a combined magnetic and structural refinement of LaCo_0.5_Rh_0.5_O_2.25_ against NPD data collected at 2 K using instrument D1B. Black ticks indicate peak positions for the crystallographic cell, red ticks the magnetic cell, and blue ticks contributions from the vanadium sample holder. (c) A selected region of NPD data collected from LaCo_0.5_Rh_0.5_O_2.25_ at 2, 100, and 175 K.

Neutron diffraction data collected from LaCo_0.5_Rh_0.5_O_2.25_ at 2 K exhibit magnetic Bragg scattering which can be indexed using a cell of the same dimensions as the crystallographic cell. Symmetry analysis reveals that there are six symmetry-distinct magnetic models which are compatible with the cell and the *I*4/*mcm* crystallographic symmetry of the phase.^22,23^ Simulating the neutron scattering from these magnetic models revealed that no single model can account for all of the observed magnetic scattering in the NPD data. A magnetic model corresponding to a C-type ordering of moments aligned in the *xy*-plane (transforming as the mM_5_^+^ irreducible representation of *I*4/*mcm*, described in magnetic space group 60.388), shown in Figure 7, accounts for all of the observed peaks except the [101]_m_ reflection (*d* = 4.47 Å). However, a G-type ordering of spins aligned along the *z*-axis (transforming as the mΓ_1_^+^ irreducible representation of *I*4/*mcm*, described in magnetic space group 140.541), shown in Figure 7, can account for the observed intensity of the [101]_m_ Bragg peak, so a magnetic model consisting of a combination of the mM_5_^+^ and mΓ_1_^+^ orderings was refined against the NPD data, in addition to a crystallographic model. During the refinement, it was assumed that only cobalt contributed to the magnetic behavior of LaCo_0.5_Rh_0.5_O_2.25_, due to the expected diamagnetism of the Rh^1+^/Rh^3+^ centers. The combined model achieved a good fit to the data collected at 2 K, as shown in Figure 7 and described in Table S7, to yield ordered moments of 1.30(11) μB and 1.19(6) μB for the mM_5_^+^ and mΓ_1_^+^ components, respectively, and a total moment of 1.76 μB for each cobalt ion.

On warming, the intensity of the [101]_m_ Bragg reflection declines in data sets collected at temperatures above 75 K, while the remaining magnetic Bragg peaks retained their intensities, as shown in Figure 7. Fitting these NPD data to the two-component magnetic model reveals that the ordered moment of the mΓ_1_^+^ component declines to zero between 75 and 125 K, while the ordered moment of the mM_5_^+^ component remains unchanged within error up to 175 K, as shown in Figure 6. Close inspection of the NPD data further revealed that the [101]_m_ Bragg peak sits on top of a broad diffuse feature, centered at the same *d*-spacing, and that the intensity of this diffuse feature declines with the intensity of the [101]_m_ reflection. Unfortunately, we were unable to measure NPD data between 175 and 300 K for operational reasons, so we could not observe the decline of the magnetic scattering intensity from the mM_5_^+^ component.

**Figure 8:**
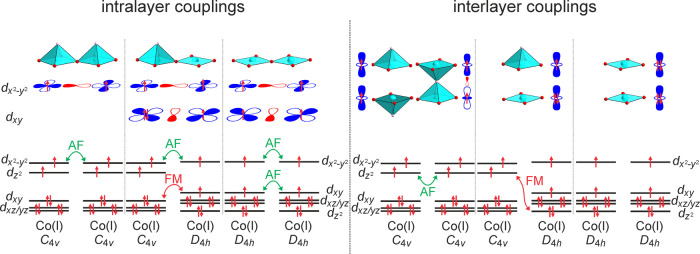
Intralayer and interlayer magnetic couplings present in LaCo_0.5_Rh_0.5_O_2.25_.

The magnetic behavior of LaCo_0.5_Rh_0.5_O_2.25_ can be rationalized by considering the magnetic coupling interactions, which exist between the *S* = 1, Co^1+^ cations (interactions with the *S* = 0, Rh^1+^ and Rh^3+^ are considered too weak to be relevant in this analysis). These Co–Co couplings can be separated into two types: intralayer couplings within the Co_0.5_Rh_0.5_O_2_ planes (i.e., the *xy*-plane of the material) and interlayer couplings between the Co^1+^ cations in adjacent Co_0.5_Rh_0.5_O_2_ layers (i.e., couplings along the *z*-axis). Considering the in-plane interactions first, there are three different couplings to consider between C_4*v*_ and D_4*h*_ coordinated Co^1+^ centers: C_4*v*_–C_4*v*_, C_4*v*_–D_4*h*_, and D_4*h*_–D_4*h*_. As shown in Figure 8, the in-plane magnetic couplings are dominated by the (3d_*x*^2^-*y*^2^_)^1^–O2p–(3d_*x*^2^-*y*^2^_)^1^ σ-type superexchange interaction, which is strongly antiferromagnetic, with only weak contributions from the π-symmetry (3d_*xy*_)–O2p–(3d_*xy*_) couplings. The resultant strong, in-plane antiferromagnetic coupling is consistent with the in-plane antiferromagnetic orderings present in both the mM_5_^+^ and mΓ_1_^+^ irreducibles.

There are also three interlayer couplings: C_4*v*_–C_4*v*_ (direct and super exchange), D_4*h*_–C_4*v*_ (direct exchange), and D_4*h*_–D_4*h*_ (direct exchange). As shown in Figure 8, when *S* = 1 Co^1+^ cations reside in D_4*h*_ coordination, all of the d-orbitals with a *z*-component are filled, so the D_4*h*_–D_4*h*_ interlayer coupling will be negligible. In contrast, the C_4*v*_–C_4*v*_ interaction will either be a strong (3d_*z*^2^_)^1^–O2p_z_–(3d_*z*^2^_)^1^ superexchange if the centers are linked by an oxide ion or (3d_*z*^2^_)^1^–(3d_*z*^2^_)^1^ direct exchange if the centers are not linked by an oxide ion. Both of these couplings are strongly antiferromagnetic, so the C_4*v*_–C_4*v*_ interlayer interaction is consistent with the mΓ_1_^+^ irreducible (G-type antiferromagnetic order). In contrast, the C_4*v*_–D_4*h*_ coupling is a (3d_*z*^2^_)^1^–(3d_*z*^2^_)^2^ direct exchange interaction that is ferromagnetic, as shown in Figure 8. Thus, the C_4*v*_–D_4*h*_ interlayer coupling is consistent with the mM_5_^+^ irreducible (C-type antiferromagnetic order). In a 1:1 disordered array of C_4*v*_ and D_4*h*_ centers, there will be twice as many C_4*v*_–D_4*h*_ interlayer couplings as C_4*v*_–C_4*v*_, consistent with the persistence of the mM_5_^+^ ordering to a higher temperature than the mΓ_1_^+^ ordering.

We, therefore, propose a model in which the interlayer and intralayer couplings between the C_4*v*_ and D_4*h*_ coordinated cobalt centers combine at 2 K to yield the mM_5_^+^ + mΓ_1_^+^ ordered model shown in Figure 7a. On warming above 75 K, the magnitude of the mΓ_1_^+^ ordered component diminishes, achieving a zero value by *T* = 125 K. On further warming, the magnitude of mM_5_^+^ component remains constant, within error up to 175 K. The lack of NPD data between 175 and 300 K makes it impossible to definitively determine the Néel temperature of the mM_5_^+^ component, but we know that there is no 3D long-range magnetic order at 300 K from the NPD data collected using the D2B instrument. It seems likely that the second magnetic transition occurs at around 200 K, the temperature at which the reciprocal of the magnetic susceptibility of the phase stops being linear with temperature. The large, nonphysical Curie constant extracted from the susceptibility data of LaCo_0.5_Rh_0.5_O_2.25_ suggests that strong, in-plane 2D magnetic correlations persist to temperatures above 300 K and further suggests that the magnetic transition associated with the loss of the magnetic scattering from the mM_5_^+^ component may be better described as a 3D-to-2D transition, hence its weak signature in the magnetic susceptibility data.

A magnetic ordering scheme arising from the addition of opposed coupling interactions (the ferromagnetic and antiferromagnetic interlayer couplings in the mM_5_^+^ and mΓ_1_^+^ components, respectively) is unusual. Typically, such opposed coupling interactions would be expected to frustrate each other leading to spin-glass behavior. In this instance, we believe that the dilution of the magnetic lattice by diamagnetic Rh^1+^ and Rh^3+^ centers gives the system enough flexibility to relieve this frustration, enabling the mM_5_^+^ and mΓ_1_^+^ components to coexist and order the *xy*- and *z*-components of the magnetic moments, respectively. As noted above, a significant amount of diffuse scattering can be observed under the [110]_m_ reflection (Figure 7), indicating that the mΓ_1_^+^ component of the magnetic order is short ranged in parts of the sample, while the displacement of the field-cooled magnetization-field isotherms, observed below 30 K, indicates a glassy component to the magnetic behavior; both features suggest that the competition/frustration between the interlayer ferromagnetic and antiferromagnetic couplings is not completely lifted.

Using the same analysis strategy, the lack of long-range magnetic order in LaNi_0.5_Rh_0.5_O_2.25_ can be rationalized by noting that the additional electron present in d^9^ Ni^1+^, compared to d^8^ Co^1+^, will fill the 3d_*z*^2^_ orbital for the C_4*v*_-coordinated Ni^1+^ and the 3d_*xy*_ orbital for the D_4*h*_-coordinated Ni^1+^ centers. As a result, all of the orbitals with a component parallel to the *z*-axis are filled, so while we would expect strong intralayer antiferromagnetic couplings in LaNi_0.5_Rh_0.5_O_2.25_, the interlayer couplings will be extremely weak, explaining the lack of long-range magnetic order in this phase.

## Conclusions

Topochemical reduction of the cation-disordered perovskite oxides LaCo_0.5_Rh_0.5_O_3_ and LaNi_0.5_Rh_0.5_O_3_ yields LaCo_0.5_Rh_0.5_O_2.25_ and LaNi_0.5_Rh_0.5_O_2.25_, respectively – compositions that appear to be selected by the stabilization provided by Rh^1+^ and Rh^3+^ centers located in square-planar and square-based pyramidal coordination sites, respectively.

The resulting arrays of Co^1+^, diluted in the perovskite framework by diamagnetic Rh^1+^/Rh^3+^ in LaCo_0.5_Rh_0.5_O_2.25_, exhibit complex magnetic order, arising from the differing d-orbital occupations of the D_4*h*_ square-planar and C_4v_ square-pyramidal coordinated Co^1+^ centers. These differing local electronic configurations mean that the C_4*v*_–C_4*v*_ interlayer coupling is antiferromagnetic, while the corresponding D_4*h*_–C_4*v*_ coupling is ferromagnetic. Unusually, the competition between these opposed interlayer magnetic couplings leads to a sequential ordering of the in-plane *xy* and interlayer *z* components of the Co spins, rather than magnetic frustration and spin glass behavior.

## Supporting Information Available

Description of the structural characterization of LaCo_0.5_Rh_0.5_O_3_ and LaNi_0.5_Rh_0.5_O_3_; a complete description of the chemical, structural, and magnetic characterization of LaCo_0.5_Rh_0.5_O_2.25_ and LaNi_0.5_Rh_0.5_O_2.25_ at 298 and 2 K (PDF)
