# Supplementary material for: Complex Magnetic Order in Topochemically Reduced Rh(I)/Rh(III) LaM0.5Rh0.5O2.25 (M = Co, Ni) Phases
Source: Inorg Chem. 2022 Sep 21;61(39):15686–92. doi: 10.1021/acs.inorgchem.2c02747 (PMC9533305; doi:10.1021/acs.inorgchem.2c02747)
Supplement: Supplementary file 1 — ic2c02747_si_001.pdf [file ic2c02747_si_001.pdf]

Supporting Information

Complex Magnetic Order in Topochemically Reduced Rh(I)/Rh(III) $LaM_{0.5}Rh_{0.5}O_{2.25}$ (M = Co, Ni) phases.

Zheying Xu[†], Pardeep K. Thakur,[‖] Tien-Lin Lee,[‖] Anna Regoutz [‡], Emmanuelle Suard[§], Inés Puente-Orench[§] and Michael A. Hayward[†*]

[†] Department of Chemistry, University of Oxford, Inorganic Chemistry Laboratory, South Parks Road, Oxford, OX1 3QR, UK.

[‡] Department of Chemistry, University College London, 20 Gordon Street, London WC1H 0AJ, UK.

[‖] Diamond Light Source Ltd., Diamond House, Harwell Science and Innovation Campus, Didcot OX11 0DE, UK.

[§] Institut Laue-Langevin - 71 avenue des Martyrs 38000 GRENOBLE - France.

*michael.hayward@chem.ox.ac.uk

Table of Contents

1. Structural Characterisation of $LaCo_{0.5}Rh_{0.5}O_3$.

Figure S1. Observed, calculated and difference plots from the structural refinement of $LaCo_{0.5}Rh_{0.5}O_3$ against SXRD data collected at room temperature.

Table S1. Parameters from the structural refinement of $LaCo_{0.5}Rh_{0.5}O_3$ against SXRD data collected at room temperature.

2. Structural Characterisation of $LaNi_{0.5}Rh_{0.5}O_3$.

Figure S2. Observed, calculated and difference plots from the structural refinement of $LaNi_{0.5}Rh_{0.5}O_3$ against SXRD data collected at room temperature.

Table S2. Parameters from the structural refinement of $LaNi_{0.5}Rh_{0.5}O_2$ against SXRD data collected at room temperature.

3. Thermogravimetric Characterisation of $LaCo_{0.5}Rh_{0.5}O_{3-x}$.

Figure S3. Thermogravimetric data collected while heating $LaCo_{0.5}Rh_{0.5}O_{3-x}$ under a 10:90 H_2:N_2 gas flow.

4. Thermogravimetric Characterisation of $LaNi_{0.5}Rh_{0.5}O_{3-x}$.

Figure S4. Thermogravimetric data collected while heating $LaNi_{0.5}Rh_{0.5}O_{3-x}$ under a 10:90 H_2:N_2 gas flow.

5. Structural Characterisation of $LaCo_{0.5}Rh_{0.5}O_{2.25}$ and $LaNi_{0.5}Rh_{0.5}O_{2.25}$ at 298K.

Table S3. Parameters from the structural refinement of $LaCo_{0.5}Rh_{0.5}O_{2.25}$ against NPD data collected at room temperature.

Table S4. Parameters from the structural refinement of $LaNi_{0.5}Rh_{0.5}O_{2.25}$ against NPD data collected at room temperature.

Table S5. Selected bond lengths and angles from the refined structures of $LaCo_{0.5}Rh_{0.5}O_{2.25}$ and $LaNi_{0.5}Rh_{0.5}O_{2.25}$.

6. HAXPES.

Figure S5. HAXPES data from $LaCo_{0.5}Rh_{0.5}O_{2.25}$, including (a) survey spectrum, (b) Rh 3*p* / O 1*s* core level spectra, and (c) valence band spectrum. In (c) the position of the Fermi level E_F at 0 eV is indicated.

7. Magnetic measurements in the presence of elemental Ni and Co impurities via the 'ferrosubtraction' method.

Figure S6. Magnetisation of $LaNi_{0.5}Rh_{0.5}O_{2.25}$ measured as a function of applied field at 300 K.

8. Structural Characterisation of $LaNi_{0.5}Rh_{0.5}O_{2.25}$ at 2K.

Figure S7. Observed calculated and difference plots from the structural refinement of $LaNi_{0.5}Rh_{0.5}O_{2.25}$ against NPD data collected at 2 K using the D1B instrument.

Table S6. Parameters from the structural refinement of $LaNi_{0.5}Rh_{0.5}O_{2.25}$ against NPD data collected at 2 K.

9. Magnetic Characterisation of $LaCo_{0.5}Rh_{0.5}O_{2.25}$.

Figure S8. Magnetisation data collected as a function of temperature using the ferrosubtraction technique.

Figure S9. Magnetisation-field data collected from $LaCo_{0.5}Rh_{0.5}O_{2.25}$ after cooling in an applied field of 50,000 Oe.

Table S7. Nuclear and magnetic structure of $LaCo_{0.5}Rh_{0.5}O_{2.25}$ determined by refinement against NPD data collected at 2K.

1. Structural Characterisation of $LaCo_{0.5}Rh_{0.5}O_3$.

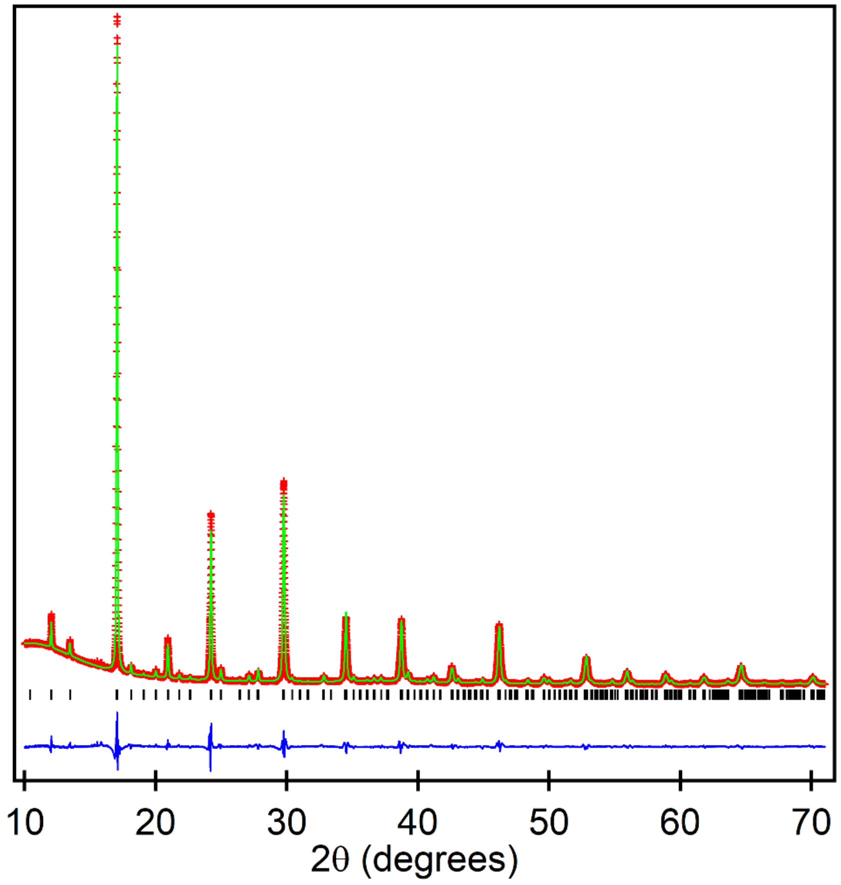

Figure S1. Observed, calculated and difference plots from the structural refinement of $LaCo_{0.5}Rh_{0.5}O_3$ against SXRD data collected at room temperature.

Atom	Site	*x*	*y*	*z*	Occupancy	Biso (Å^2)
La	4*c*	04710(1)	¼	0.5055(2)	1	0.65(1)
Co/Rh	4*b*	0	0	½	0.5/0.5	0.10(1)
O(1)	4*c*	0.002(1)	¼	0.403(2)	1	0.30(1)
O(2)	8*d*	0.210(1)	0.022(1)	0.801(1)	1	0.30(1)
$LaCo_{0.5}Rh_{0.5}O_3$ space group *Pnma* (#62)<br>*a* = 5.5722(1) Å, *b* = 7.8837(3) Å, *c* = 5.5860(2) Å, volume = 245.39(1) Å^3<br>Formula weight = 267.83 g mol^{-1}, Z = 2<br>Radiation source: Synchrotron X-ray, λ = 0.8268 Å<br>Temperature: 298 K<br>R_p = 2.71 %, $_wR_p$ = 4.04 %, R_{Bragg} = 1.31 %						

Table S1. Parameters from the structural refinement of $LaCo_{0.5}Rh_{0.5}O_3$ against SXRD data collected at room temperature.

2. Structural Characterisation of $LaNi_{0.5}Rh_{0.5}O_3$.

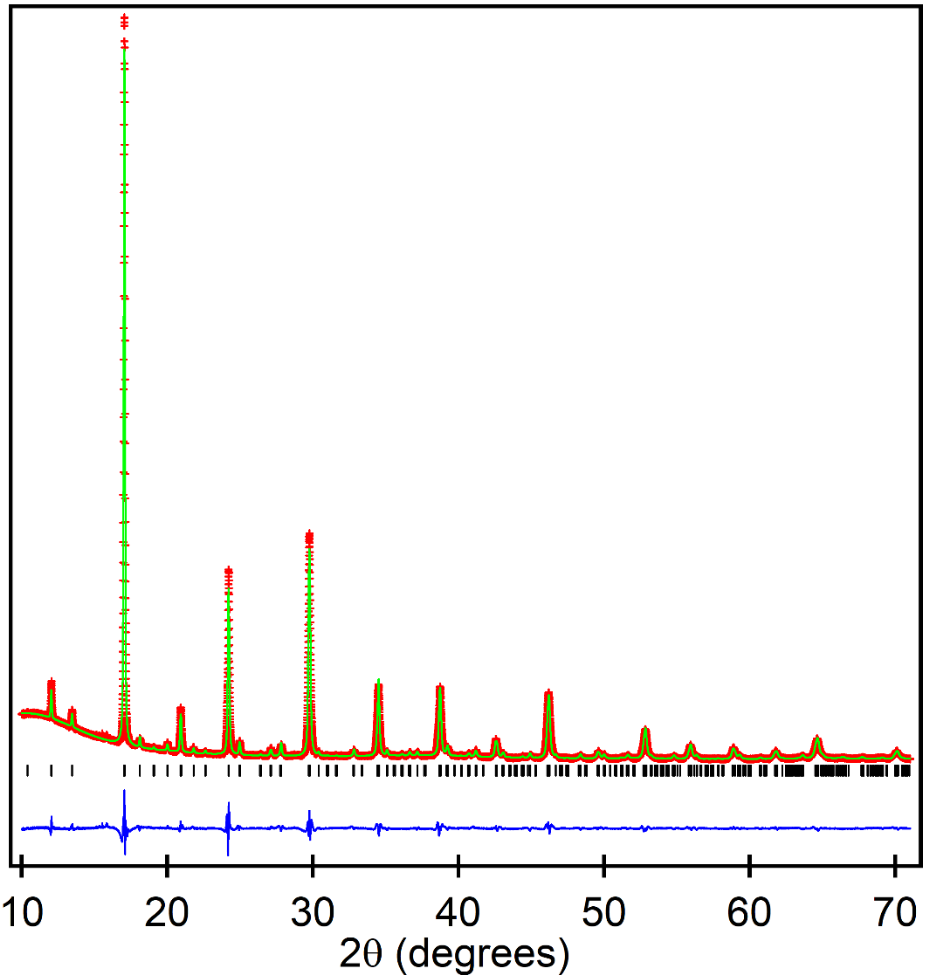

Figure S2. Observed, calculated and difference plots from the structural refinement of $LaNi_{0.5}Rh_{0.5}O_3$ against SXRD data collected at room temperature.

Atom	Site	*x*	*y*	*z*	Occupancy	Biso (Å^2)
La	4*c*	04635(1)	¼	0.5009(9)	1	0.65(1)
Ni/Rh	4*b*	0	0	½	0.5/0.5	0.10(1)
O(1)	4*c*	0.023(1)	¼	0.385(2)	1	0.30(1)
O(2)	8*d*	0.224(1)	0.041(1)	0.783(1)	1	0.30(1)
$LaNi_{0.5}Rh_{0.5}O_3$ space group *Pnma* (#62)<br>*a* = 5.5531(1) Å, *b* = 7.8452(3) Å, *c* = 5.5640(1) Å, volume = 242.40(1) Å^3<br>Formula weight = 267.71 g mol^{-1}, Z = 2<br>Radiation source: Synchrotron X-ray, λ = 0.8268 Å<br>Temperature: 298 K<br>R_p = 2.29 %, $_wR_p$ = 3.61 %, R_{Bragg} = 1.03 %						

Table S2. Parameters from the structural refinement of $LaNi_{0.5}Rh_{0.5}O_2$ against SXRD data collected at room temperature.

3. Thermogravimetric Characterisation of $LaCo_{0.5}Rh_{0.5}O_{3-x}$.

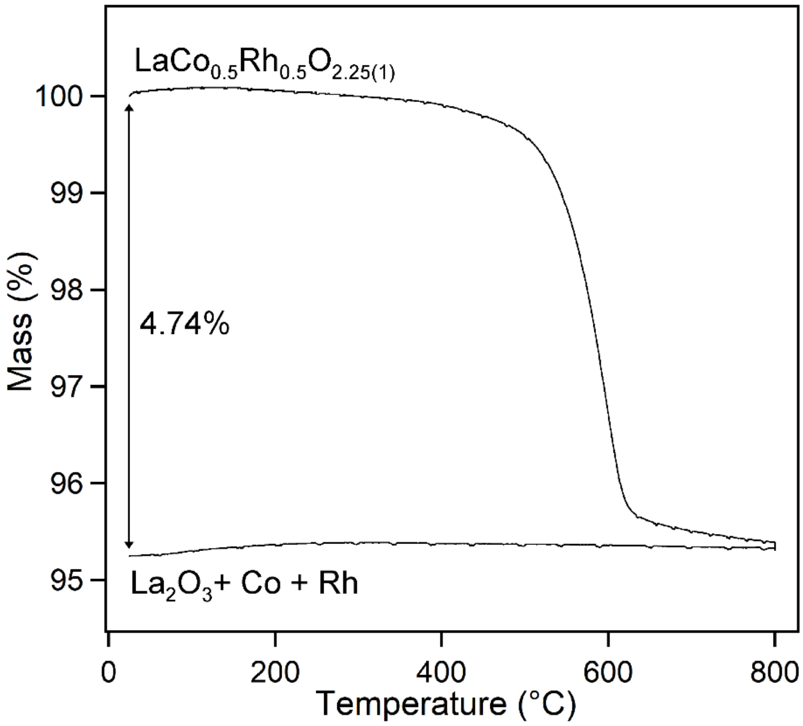

Figure S3. Thermogravimetric data collected while heating $LaCo_{0.5}Rh_{0.5}O_{3-x}$ under a 10:90 H_2:N_2 gas flow. X-ray powder diffraction data indicates the sample decomposed to a mixture of La_2O_3, Co and Rh, losing 4.74% mass. This indicates an initial composition of $LaCo_{0.5}Rh_{0.5}O_{2.25(1)}$.

4. Thermogravimetric Characterisation of $LaNi_{0.5}Rh_{0.5}O_{3-x}$.

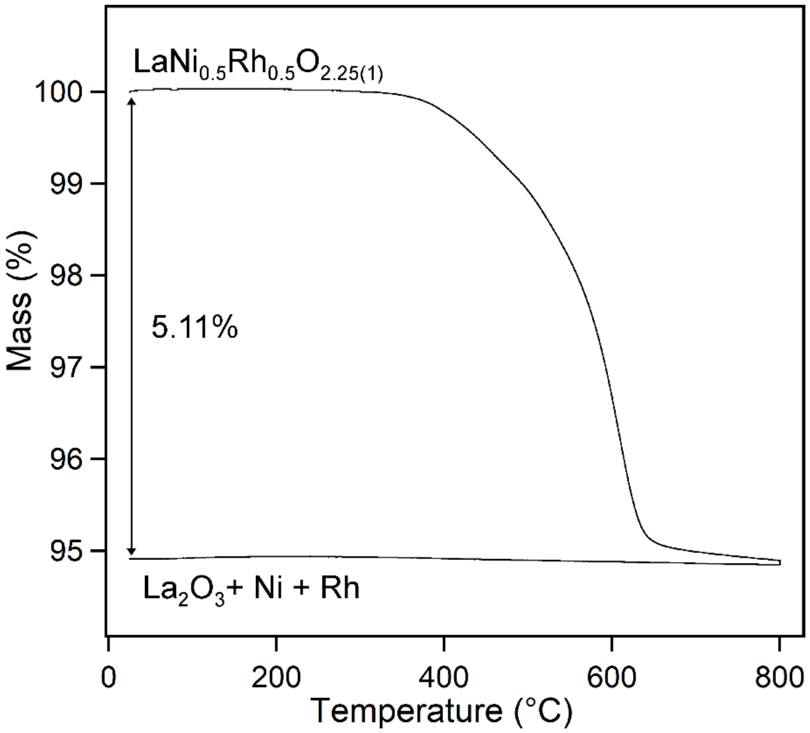

Figure S4. Thermogravimetric data collected while heating $LaNi_{0.5}Rh_{0.5}O_{3-x}$ under a 10:90 H_2:N_2 gas flow. X-ray powder diffraction data indicates the sample decomposed to a mixture of La_2O_3, Ni and Rh, losing 5.11% mass. This indicates an initial composition of $Lani_{0.5}Rh_{0.5}O_{2.25(1)}$ when the 9.2 weight % $LaNi_{0.5}Rh_{0.5}O_3$ observed in NPD data is taken into account.

5. Structural Characterisation of $LaCo_{0.5}Rh_{0.5}O_{2.25}$ and $LaNi_{0.5}Rh_{0.5}O_{2.25}$ at 298K.

Atom	Site	*x*	*y*	*z*	Occupancy	B_{iso} (Å^2)
La	4*b*	½	0	¼	1	0.32(1)
Co/Rh	4*c*	0	0	0	0.5/0.5	0.43(1)
O (1)	8*h*	0.2139(2)	0.2860(2)	0	1	0.83(2)
O (2)	4*a*	0	0	¼	0.25(1)	0.83(2)
$LaCo_{0.5}Rh_{0.5}O_{2.25(1)}$ - space group *I*4/*mcm* (#140)<br>*a* = 5.6492(2) Å, *c* = 7.3374(7) Å,<br>Volume = 234.16(3) Å^3<br>Formula weight: 255.83 g mol^{-1}, Z = 4						
Radiation source: Neutron, λ = 1.5942 Å<br>Temperature: 298 K<br>*wRp* = 2.77%; *Rp* = 2.18%, R_{Bragg} 1.01%						

Table S3. Parameters from the structural refinement of $LaCo_{0.5}Rh_{0.5}O_{2.25}$ against NPD data collected at room temperature.

Atom	Site	*x*	*y*	*z*	Occupancy	B_{iso} (Å^2)
La	4*b*	½	0	¼	1	0.43(1)
Ni/Rh	4*c*	0	0	0	0.5/0.5	0.52(1)
O (1)	8*h*	0.215(2)	0.284(2)	0	1	0.91(2)
O (2)	4*a*	0	0	¼	0.24(2)	0.91(2)
$LaNi_{0.5}Rh_{0.5}O_{2.25(1)}$ - space group *I*4/*mcm* (#140)<br>*a* = 5.6407(1) Å, *c* = 7.1794(7) Å,<br>Volume = 228.44(2) Å^3<br>Formula weight: 255.71 g mol^{-1}, Z = 4<br>90.2(3) weight percent<br>R_{Bragg} 1.03%						
$LaNi_{0.5}Rh_{0.5}O_3$ - space group *Pnma* (#62)<br>*a* = 5.514(7) Å, *b* = 7.801(9) *c* = 5.538(5) Å,<br>Volume = 238.2(4) Å^3<br>Formula weight: 267.71 g mol^{-1}, Z = 4<br>9.8(3) weight percent						
Radiation source: Neutron, λ = 1.5942 Å<br>Temperature: 298 K<br>*wRp* = 1.66%; *Rp* = 1.26%						

Table S4. Parameters from the structural refinement of $LaNi_{0.5}Rh_{0.5}O_{2.25}$ against NPD data collected at room temperature.

		Bond length (Å)	
Cation	Anion	$LaCo_{0.5}Rh_{0.5}O_{2.25}$	$LaNi_{0.5}Rh_{0.5}O_{2.25}$
M/Rh	O(1) × 4	2.018(1)	2.009(11)
M/Rh	O(2) × (2 × ¼)	1.834(1)	1.795(1)
La	O(1) × 4	2.930(1)	2.893(9)
La	O(1) × 4	2.507(1)	2.485(8)
La	O(2) × (4 × ¼)	2.825(1)	2.820(1)
		Bond angle (°)	
O(1)-O(1)-O(1)		163.59(3) °	164.2(3) °

Table S5. Selected bond lengths and angles from the refined structures of $LaCo_{0.5}Rh_{0.5}O_{2.25}$ and $LaNi_{0.5}Rh_{0.5}O_{2.25}$.

6. HAXPES.

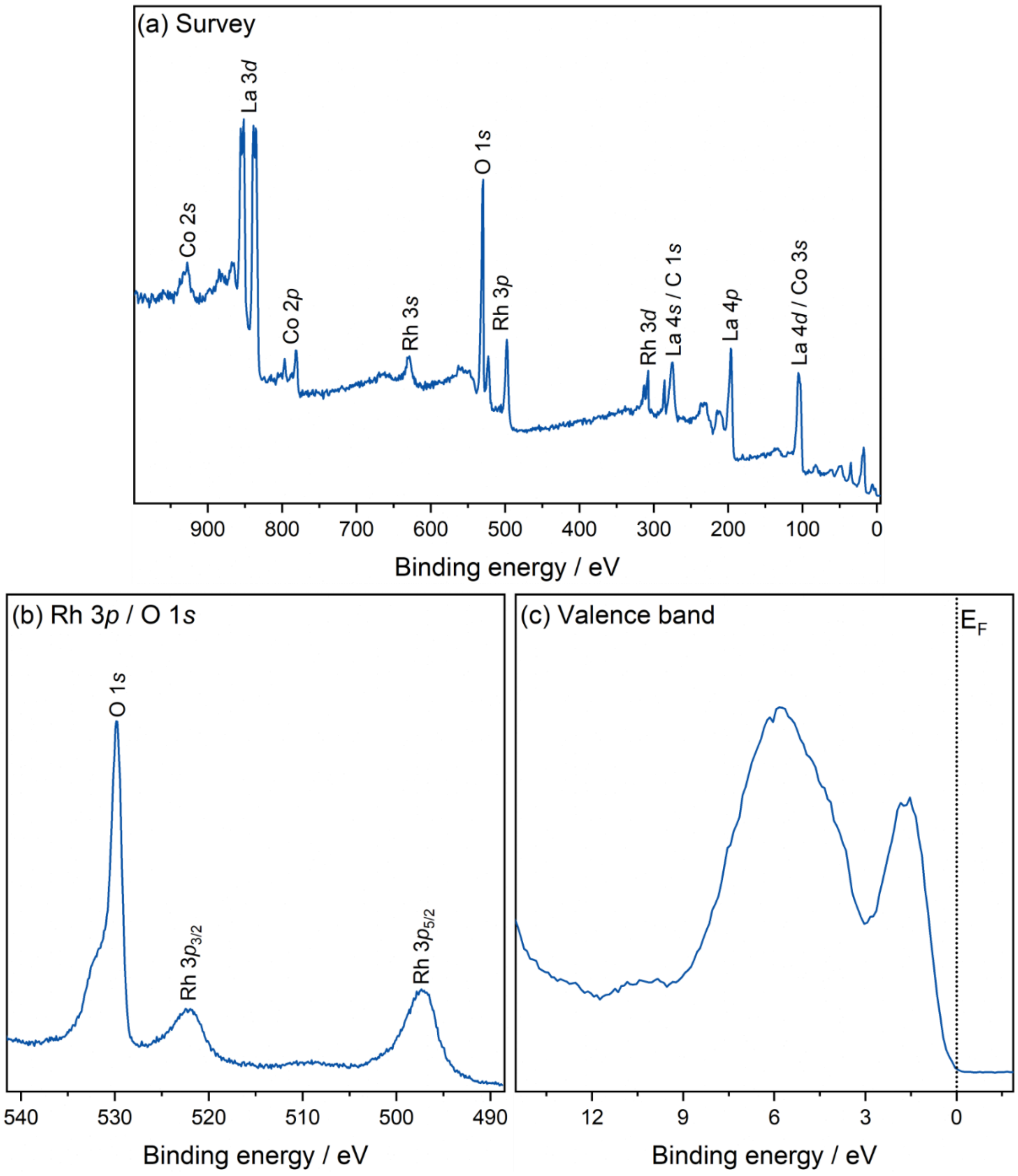

Figure S5. HAXPES data from $LaCo_{0.5}Rh_{0.5}O_{2.25}$, including (a) survey spectrum, (b) Rh 3*p* / O 1*s* core level spectra, and (c) valence band spectrum. In (c) the position of the Fermi level E_F at 0 eV is indicated.

7. Magnetic measurements in the presence of elemental Ni and Co impurities via the 'ferrosubtraction' method.

Procedure used to measure the magnetization of samples containing elemental nickel: The magnetization of elemental Ni and Co is observed to saturate in applied magnetic fields of more than 2 T. Thus the paramagnetic susceptibility of a bulk sample can be measured in the presence of elemental Ni and Co impurities by measuring the gradient of magnetization-field isotherms in applied fields larger than 2 T. As shown in Figure S6.

To this end the magnetization of samples was measured in a series of 5 fields between 3 T and 5 T. The magnetization vs. field data were fitted to a linear function, the gradient of which is the paramagnetic susceptibility of the bulk sample and the intercept is the saturated ferromagnetic moment of the sample. Data points with large errors were excluded from fits. All fits had at least 4 data points. This procedure was repeated at 5 K intervals between 5 K and 300 K to measure the temperature dependent susceptibility of samples.

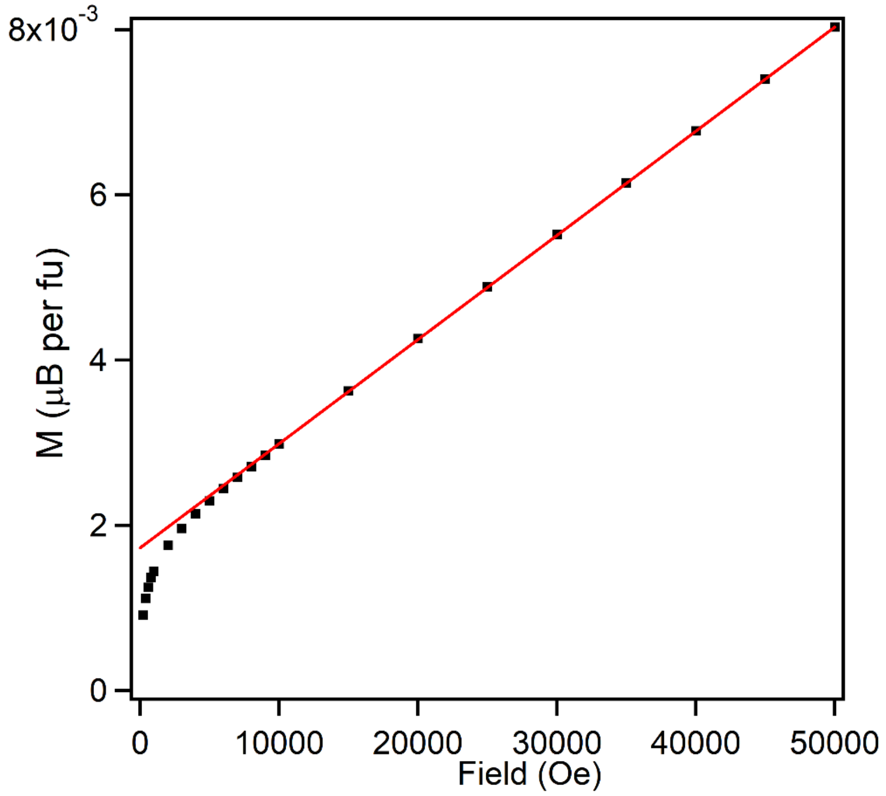

Figure S6. Magnetisation of $LaNi_{0.5}Rh_{0.5}O_{2.25}$ measured as a function of applied field at 300 K. A linear fit to high-field region (H > 25000 Oe) yields a gradient which is the paramagnetic susceptibility of the sample, and an intercept which is the saturated ferromagnetic moment of the sample.

8. Structural Characterisation of $LaNi_{0.5}Rh_{0.5}O_{2.25}$ at 2K.

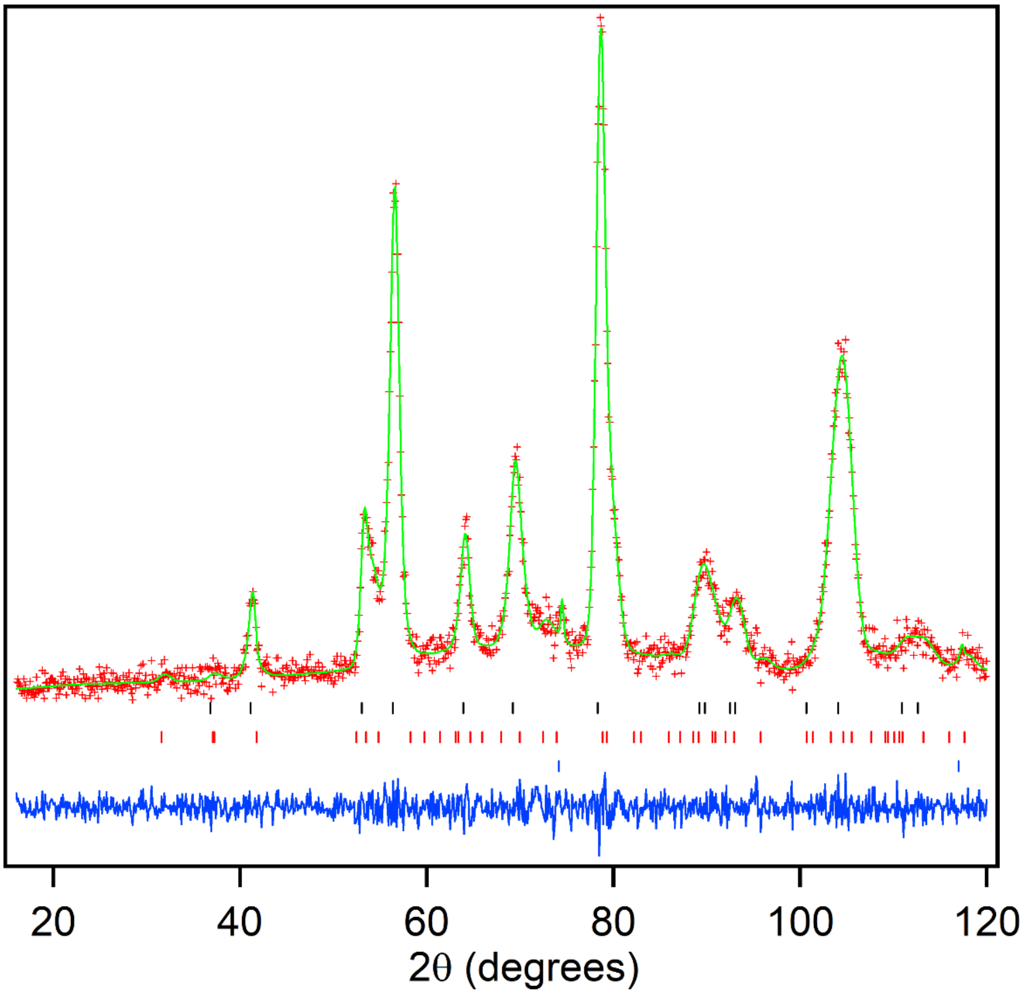

Figure S7. Observed calculated and difference plots from the structural refinement of $LaNi_{0.5}Rh_{0.5}O_{2.25}$ against NPD data collected at 2 K using the D1B instrument. Black and red and blue tick marks indicated peak positions for the majority phase, a $LaNi_{0.5}Rh_{0.5}O_3$ secondary phase and contributions from the vanadium sample holder, respectively.

Atom	Site	*x*	*y*	*z*	Occupancy	B_{iso} (Å^2)
La	4*b*	½	0	¼	1	0.38(1)
Ni/Rh	4*c*	0	0	0	0.5/0.5	0.51(2)
O (1)	8*h*	0.217(3)	0.282(3)	0	1	0.86(2)
O (2)	4*a*	0	0	¼	0.24(1)	0.86(2)
$LaNi_{0.5}Rh_{0.5}O_{2.25(1)}$ - space group *I*4/*mcm* (#140)<br>*a* = 5.638(1) Å, *c* = 7.178(1) Å,<br>Volume = 228.16(2) Å^3<br>Formula weight: 255.71 g mol^{-1}, Z = 4<br>85.2(3) weight percent<br>R_{Bragg} 1.03%						
$LaNi_{0.5}Rh_{0.5}O_3$ - space group *Pnma* (#62)<br>*a* = 5.507(8) Å, *b* = 7.798(7) *c* = 5.534(4) Å,<br>Volume = 237.6(4) Å^3<br>Formula weight: 267.71 g mol^{-1}, Z = 4<br>14.8(3) weight percent						
Radiation source: Neutron, λ = 2.52 Å<br>Temperature: 2 K<br>*wRp* = 2.36%; *Rp* = 1.76%						

Table S6. Parameters from the structural refinement of $LaNi_{0.5}Rh_{0.5}O_{2.25}$ against NPD data collected at 2 K.

9. Magnetic Characterisation of $LaCo_{0.5}Rh_{0.5}O_{2.25}$.

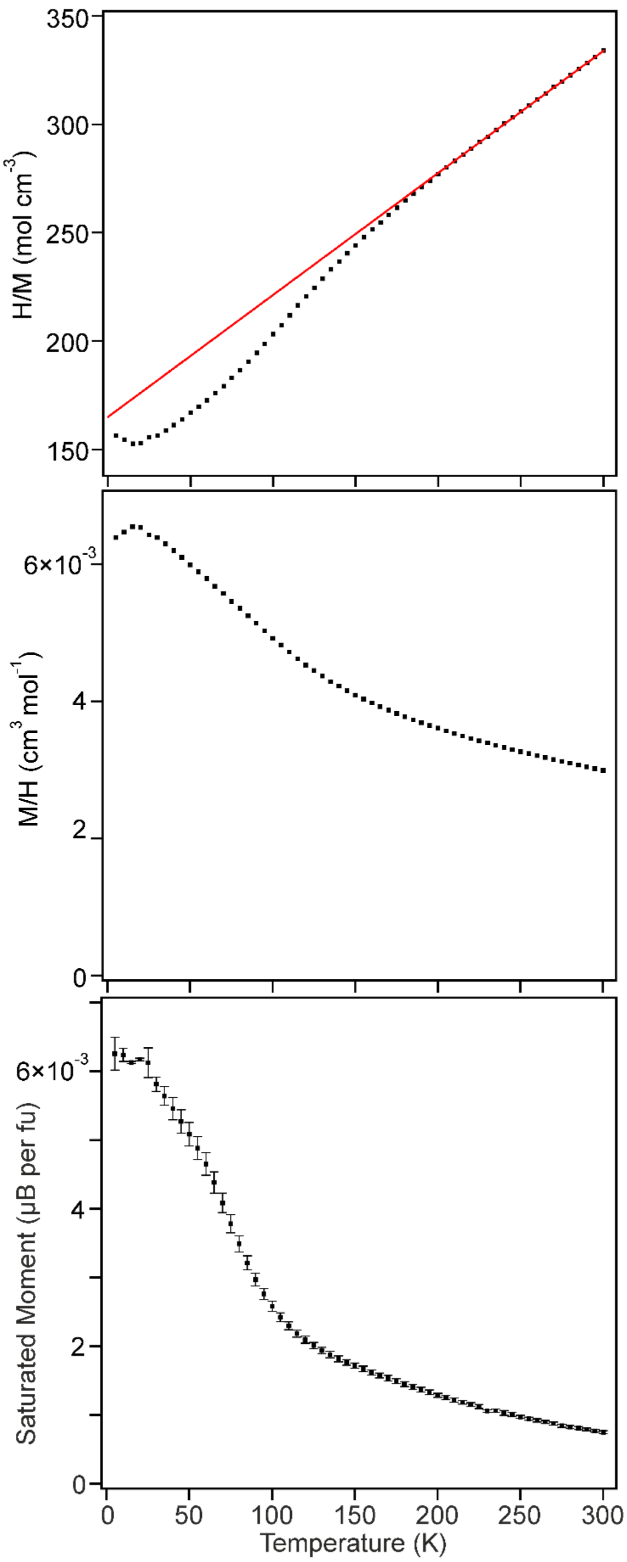

Figure S8. Magnetisation data collected as a function of temperature using the ferrosubtraction technique from $LaCo_{0.5}Rh_{0.5}O_{2.25}$. Top Panel shows fit of paramagnetic susceptibility to the Curie-Weiss Law in the range 210 < T/K < 300. Middle panel shows paramagnetic susceptibility. Bottom panel shows saturated Ferromagnetic moment.

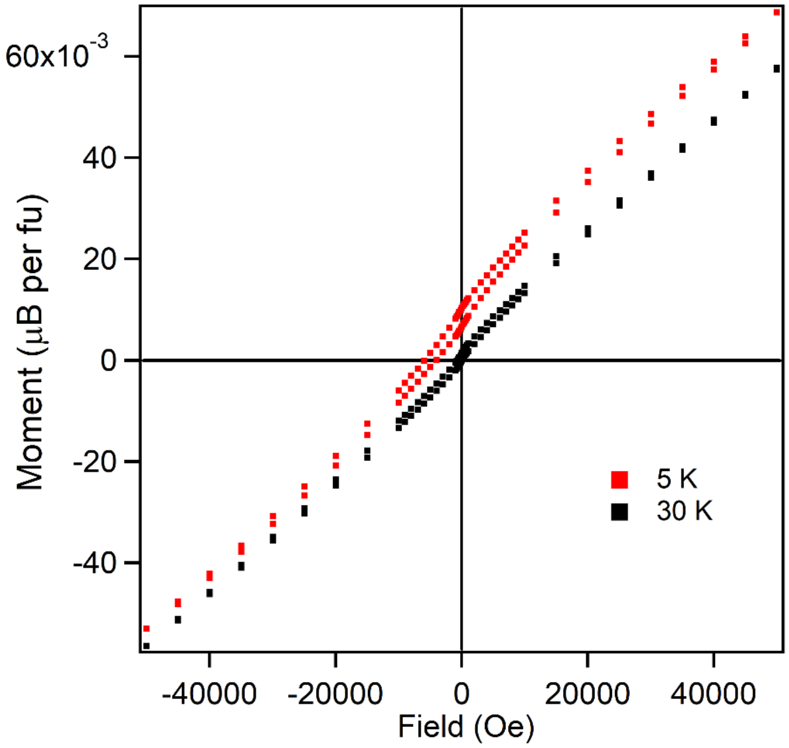

Figure S9. Magnetisation-field data collected from $LaCo_{0.5}Rh_{0.5}O_{2.25}$ after cooling in an applied field of 50,000 Oe.

Atom	Site	*x*	*y*	*z*	Occupancy	B_{iso} (Å^2)
La	4*b*	½	0	¼	1	0.22(1)
Co/Rh	4*c*	0	0	0	0.5/0.5	0.41(1)
O (1)	8*h*	0.2139(2)	0.2860(2)	0	1	0.63(2)
O (2)	4*a*	0	0	¼	0.25(1)	0.63(2)
$LaCo_{0.5}Rh_{0.5}O_{2.25(1)}$ - space group *I*4/*mcm* (#140)<br>*a* = 5.626(1) Å, *c* = 7.309(1) Å,<br>Volume = 231.3(1) Å^3<br>Formula weight: 255.83 g mol^{-1}, Z = 4						
Atom	*x*	*y*	*z*	m_x/μ_B	m_y/μ_B	m_z/μ_B
Co(1)	0	0	0	1.30(11)	0	1.19(6)
Co(2)	½	½	0	-1.30(11)	0	-1.19(6)
Co(3)	0	0	½	1.30(11)	0	-1.19(6)
Co(4)	½	½	½	-1.30(11)	0	1.19(6)
Radiation source: Neutron, λ = 2.52 Å<br>Temperature: 2 K<br>*wRp* = 2.17%; *Rp* = 1.61%, R_{Bragg} 1.03%						

Table S7. Nuclear and magnetic structure of $LaCo_{0.5}Rh_{0.5}O_{2.25}$ determined by refinement against NPD data collected at 2K. The magnetic model is a combination of an mM_5^+ irreducible representation describing the m_x component of the ordered moments (magnetic space group 60.432) and an $m\Gamma_1^+$ irreducible representation describing the m_z combination of the ordered moments (magnetic space group 140.541). The cobalt centres in the magnetic model have an occupancy of 0.5.
